# A Bayesian method for calculating real-time quantitative PCR calibration curves using absolute plasmid DNA standards

**DOI:** 10.1186/1471-2105-9-120

**Published:** 2008-02-25

**Authors:** Mano Sivaganesan, Shawn Seifring, Manju Varma, Richard A Haugland, Orin C Shanks

**Affiliations:** 1 U.S. Environmental Protection Agency, Office of Research and Development, National Risk Management Research Laboratory, 26 West Martin Luther King Drive, Cincinnati, OH 45268, USA; 2 U.S. Environmental Protection Agency, Office of Research and Development, National Exposure Research Laboratory, 26 West Martin Luther King Drive, Cincinnati, OH 45268, USA

## Abstract

**Background:**

In real-time quantitative PCR studies using absolute plasmid DNA standards, a calibration curve is developed to estimate an unknown DNA concentration. However, potential differences in the amplification performance of plasmid DNA compared to genomic DNA standards are often ignored in calibration calculations and in some cases impossible to characterize. A flexible statistical method that can account for uncertainty between plasmid and genomic DNA targets, replicate testing, and experiment-to-experiment variability is needed to estimate calibration curve parameters such as intercept and slope. Here we report the use of a Bayesian approach to generate calibration curves for the enumeration of target DNA from genomic DNA samples using absolute plasmid DNA standards.

**Results:**

Instead of the two traditional methods (classical and inverse), a Monte Carlo Markov Chain (MCMC) estimation was used to generate single, master, and modified calibration curves. The mean and the percentiles of the posterior distribution were used as point and interval estimates of unknown parameters such as intercepts, slopes and DNA concentrations. The software WinBUGS was used to perform all simulations and to generate the posterior distributions of all the unknown parameters of interest.

**Conclusion:**

The Bayesian approach defined in this study allowed for the estimation of DNA concentrations from environmental samples using absolute standard curves generated by real-time qPCR. The approach accounted for uncertainty from multiple sources such as experiment-to-experiment variation, variability between replicate measurements, as well as uncertainty introduced when employing calibration curves generated from absolute plasmid DNA standards.

## Background

The goal for many real-time quantitative PCR (qPCR) assays with clinical, forensic, or environmental applications is to develop a standardized method that can be implemented on an inter-laboratory scale. Real-time qPCR assays are ideal for such applications due to high levels of precision, specificity, and sensitivity. Real-time PCR allows for the continuous monitoring of PCR product production as the reaction occurs. Under ideal conditions these products accumulate exponentially in the reactions, i.e. their quantities double with each thermal cycle. Thus, real-time qPCR can be applied to determine a fixed threshold where the accumulation of PCR product is first significantly detectable over a real-time measurement background signal [for review see [1]]. The fractional cycle number where PCR product accumulation passes this fixed threshold is called the threshold cycle (C_T_) [2]. qPCR is based on the theoretical premise that there is a log-linear relationship between the starting amount of DNA target in the reaction and the C_T _value that is obtained. The C_T _value can then be used to estimate the initial concentration of a DNA target from an unknown sample.

### Relative and Absolute Quantification with Real-Time qPCR

Two general strategies are often used to estimate DNA concentration from C_T _values including relative and absolute approaches [3]. A relative quantification approach measures the change in target DNA concentration relative to another reference sample. This approach is ideal in gene expression studies where the goal is to measure the regulation of a gene in response to a particular treatment. However, a relative approach can be limiting for qPCR applications designed to quantify DNA targets with no clear connections to a reference target such as assays where the DNA target is from an uncharacterized microorganism. Relative quantification based qPCR methods can also be difficult to apply on an inter-laboratory scale for the enumeration of DNA targets from highly variable, complex, and poorly described sample matrices such as gastrointestinal and environmental samples [4].

Absolute quantification is another widely used strategy. Absolute quantification is achieved by using a standard curve, constructed by amplifying known amounts of target DNA in a parallel set of reactions [5]. Absolute quantification requires that the exact quantity of a standard is determined by independent means using spectrophotometry or an intercalating dye such as PicoGreen^® ^[6]. For bacterial DNA targets, genomic DNA from pure cell cultures is preferred. Cultivated bacterial cells can be isolated and counted to provide a conversion factor between mass of genomic DNA, copies of target DNA, and number of cells. However, this practice imposes a substantial restriction on the development of real-time qPCR methods targeting bacterial genes because an estimated 99% of the microbial diversity on the planet has not been cultivated [7-10]. When a DNA target originates from an uncultivated microorganism, plasmid DNA standards are often used. Plasmid preparations are advantageous because these preparations generate high quality, pure, and concentrated standards that can be independently quantified and converted to number of copies of target DNA. For absolute quantification approaches, an assumption must be made that plasmid and genomic DNA amplify with the same efficiency. Factors such as DNA stability, base composition, secondary structure, and presence of complex mixtures of non-target DNA could significantly alter amplification performance. A limited number of strategies have been used in an attempt to equilibrate these two types of DNA for real-time qPCR applications such as treating genomic DNA with a cocktail of restriction enzymes and DNA ultrasonication [11]. However, many studies simply assume that there are no differences.

In addition to the uncertainty associated with amplification of plasmid versus genomic DNA targets, there are a number of other sources of variability to consider when generating a calibration curve from absolute standards. Uncertainty can arise within and between experiments from numerous sources such as inconsistencies in quality of reagents, pipet calibration, as well as dilution preparation and storage of standards. Any of these factors could significantly alter C_T _measurements from experiment to experiment. Therefore, estimation of uncertainty becomes critical to account for sources of variability and make reasonable estimates of calibration curve parameters.

### Estimating DNA Concentrations from C_T _Values and Propagation of Uncertainty

Simple linear regression is commonly used to estimate DNA concentration from an unknown sample where the standard calibration model is developed with a DNA concentration (ie. plasmid copy number) and associated C_T _measurements. Typically four to five known DNA concentrations are selected and then triplicate C_T _measurements are taken at each DNA concentration to fit a calibration curve. The fitted curve is then used to estimate the mean DNA concentrations of unknown samples.

Widely used standard methods for generating calibration curves from absolute standards and estimating DNA concentration include the classical and inverse approaches. The classical approach assumes DNA concentration as the independent variable and C_T _measurement as the dependent variable. Usually each experiment is repeated three to four times, with three replicates within each experiment. Even though triplicate C_T _measurements are taken at each DNA concentration of each experiment, the average of the C_T _measurements is commonly used to fit the calibration curve [12]. The corresponding regression model is given by:

(1) $$\begin{array}{l} {\text{Y}}_{\text{i}}\sim \text{N}(\mu_{\text{i}},\sigma^{2}),\\ \begin{array}{cc} \mu_{\text{i}}=\alpha +\beta *\log_{10}({\text{X}}_{\text{i}}), & \text{i}=1,2,...,\text{n} \end{array} \end{array}$$

where, n is the total number of DNA concentrations, Y_i _is the average of the C_T _measurements at the ith DNA concentration, X_i _is the corresponding DNA concentration, *α *and *β *are regression coefficients and *σ*^2 ^is the random error variance. For an unknown mean value of log_10_(X), say log_10_(X_0_), a Y value, say Y_0 _is observed. The classical method uses Y_0 _to estimate log_10_(X_0_) by:

(2) $$\log_{10}({\hat{X}}_{0})=\frac{Y_{0}-\hat{\beta }}{\hat{\alpha }}$$

where, $\hat{\alpha }$ and $\hat{\beta }$ are least squares estimates of *α *and *β*, respectively. Finding the standard deviation of log_10_(${\hat{X}}_{0}$) is not a simple statistical problem as it is a non-linear function of the estimated intercept and slope parameters. Thus for given X, a 100(1-*α*)% confidence interval is constructed for $\hat{Y}(=\hat{\alpha }+\hat{\beta }\log_{10}(X))$ first, as it is a linear function of intercept and slope parameters. The formula for this interval is given by:

(3) $$\hat{Y}\pm t_{n-2}(\alpha /2)\sqrt{(\frac{1}{n}+\frac{{\left(\bar{Z}-\log_{10}X\right)}^{2}}{\sum (Z_{i}-\bar{Z}){]}^{2}})\cdot \frac{\sum {\left(Y_{i}-\bar{Y}\right)}^{2}}{n-2}}$$

where Z_i _= log_10_(X_i_). Then the corresponding fiducial interval is reported as the confidence interval for X (given Y).

Another approach in practice is to estimate the unknown DNA concentration using triplicate C_T _measurements from one experiment to obtain the calibration curve [13]. The corresponding regression model for replicated data is then given by:

(4) $$\begin{array}{l} {\text{Y}}_{\text{ij}}\sim \text{N}(\mu_{\text{ij}},\sigma^{2}),\\ \begin{array}{cc} \mu_{\text{ij}}=\alpha +\beta *\log_{10}({\text{X}}_{\text{i}}), & \text{i}=1,2,...,\text{n};\text{ j}=1,2,3 \end{array} \end{array}$$

where, Y_ij _is the jth C_T _measurement of ith DNA concentration. Except for more data points, the above regression model is same as the model given by Equation (1). The same least squares method is used to estimate the model parameters and then equation (2) is used to estimate unknown concentrations.

The inverse method to estimate the unknown DNA concentration assumes a simple linear regression of X on Y on the same replicated data given by equation (4) in the classical method [14]. The inverse regression model is given by:

(5) $$\begin{array}{l} \log_{10}({\text{X}}_{\text{i}})\sim \text{N}(\delta_{\text{ij}},\sigma_{0}^{2}),\\ \begin{array}{cc} \delta_{\text{ij}}=\delta_{0}+\delta_{1}*{\text{Y}}_{\text{ij}}, & \text{i}=1,2..\text{n};\text{ j}=1,2,3 \end{array} \end{array}$$

The inverse estimator of X_0 _is given by:

(6) $$\log_{10}({\hat{X}}_{0})={\hat{\delta }}_{0}+{\hat{\delta }}_{1}\cdot Y_{0}$$

where, ${\hat{\delta }}_{0}$ and ${\hat{\delta }}_{1}$ are respectively the least squares estimates of *δ*_0 _and *δ*_1_. An approximate 100(1-*α*)% confidence interval is given by :

(7) $$\log_{10}({\hat{X}}_{0})\pm t_{n-2}(\alpha /2)\sqrt{(\frac{1}{n}+\frac{{\left(\bar{Y}-Y_{0}\right)}^{2}}{\sum {\left(Y_{i}-\bar{Y}\right)}^{2}})\cdot \frac{\sum {\left(Z_{i}-\bar{Z}\right)}^{2}}{n-2}}$$

An alternative approach to the classical and the inverse approaches is a Bayesian method using a Monte Carlo Markov Chain (MCMC) simulation technique. A detailed description of this method to generate a master calibration curve is discussed in the results and discussion section. Bayesian approaches have been employed in many molecular applications and have been particularly useful for microarray data analyses to account for multiple sources of uncertainty arising from experimental variation, background noise, and the use of multiple hybridization probes with different lengths and base pair compositions [15,16]. Bayesian principles have also been used to model PCR amplification curves [17] and characterize the relationship between fluorescence chemistry and determination of C_T _values during real-time detection [18].

Here we report the use of a Bayesian approach to generate calibration curves for the enumeration of target DNA from genomic DNA samples using absolute plasmid DNA standards. Calibration curves were generated from three independent real-time qPCR assays (Btheta, Entero1 and Entero2) using both genomic and plasmid DNA standards to test the assumption that both DNA types generate similar calibration curves. Finally, a calibration curve was generated for an additional real-time qPCR assay (HF183) where only a plasmid absolute standard was available. To account for potential differences in amplification performance between the plasmid standards and genomic DNA target from unknown samples, MCMC simulations were used to estimate the mean difference in slope and intercept from fitted curve equations for plasmid and genomic DNA produced from assays Btheta, Entero1, and Entero2. Using the same MCMC approach, these differences were applied to the plasmid DNA derived calibration curve for HF183. The modified calibration curve was then used to estimate DNA concentration from several unknown samples. The MCMC approach was ideal because it not only accounted for observed mean differences in plasmid and genomic DNA standards, but also propagated intra- and inter-assay variation.

## Results and discussion

### Bayesian Simulation Method

The Bayesian approach to statistical modeling is based on the premise that the uncertainty about unknown quantities, such as the parameters in a model, is described by a probability distribution; more precisely by a conditional probability distribution given all that is known, including the data as it becomes available. Initially, i.e., prior to obtaining the data, the uncertainty about the parameters are described by what is known as the prior distribution of the parameters, which probabilistically summarizes any available prior information about the parameters. Once the data is obtained and a suitable model for the observed data is chosen, the likelihood function of the parameters summarizing the information in the data can be mathematically expressed. The prior distribution is then combined with the likelihood via Bayes theorem, to obtain what is known as the posterior distribution of the parameters. The posterior distribution is a probabilistic expression of the (remaining) uncertainty about the parameters, after incorporating the available prior information and the information contained in the data. It is therefore the posterior distribution that forms the basis for Bayesian inference about the unknown parameters.

Typically, summaries of the posterior distribution such as the mean and the percentiles are used as point and interval estimates of an unknown parameter. In this paper, we use the term Bayesian credible interval (BCI) to refer to the interval with equal tail probability on either side under the posterior distribution. Closed form solutions for these quantities are usually not available, but, in most cases, MCMC methods [19-21] can be used to numerically compute the desired summaries of the posterior distribution. MCMC methods first use an iterative algorithm to generate a sequence of draws from a suitable Markov chain. Drawing a sufficiently long sequence, referred to as the burn-in phase, typically ensures convergence. Convergence is needed for the estimates of unknown model parameters. Examining the trace plots of the sample values of a model parameter provides evidence of when the simulation appears to have stabilized. Subsequent draws, after the burn-in phase, is a (Monte Carlo) sample from the posterior distribution, which can be used to calculate desired summaries of the posterior distribution.

The MCMC calculations in this study were done using the publicly available software WinBUGS [22]. Often, prior information about an unknown parameter may not be available. In such cases, standard non-informative prior distributions, i.e., probability distributions which contain little or no information about the parameters, are used, resulting in posterior distributions that are dominated by the likelihood. Some of the advantages of the Bayesian approach via MCMC are that it is capable of fitting models accounting for different sources of variability, and it allows for the appropriate processing of uncertainty when inference about complex functions of the model parameters are of interest. In such cases, the traditional methods tend to use approximations based on the basic summary values, i.e., estimates of model parameters and their standard errors, to obtain the inference, whereas the Bayesian approach via MCMC accurately evaluates the inference using the joint posterior distribution of the parameters. The Bayesian approach, however, also requires the specification of distributions of additional quantities in the models, as well as extensive simulation to fit them.

### Developing a Calibration Curve from a single qPCR experiment

A Bayesian approach was used to estimate the calibration curve parameters. To estimate X_0_, we use all the triplicate C_T _measurements from a single experiment to fit the calibration curve. The simple linear regression model given by the equation (3) was used here to fit the data. As no prior information is assumed for the model parameters *α*, *β *and *σ*^2^, the following diffused prior distributions are used to estimate these model parameters:

*α*, *β *~ N (0, 10^6^)

*σ*^2 ^~ Inv. Gamma(.0001,.0001).

These are essentially flat priors (i.e the prior essentially assigns equal weights to all possible values of the parameters), and hence would lead to posteriors dominated by likelihood. According to Bayes theorem, the posterior distribution of the model parameters, *α*, *β *and *σ*^2^, given the data *y*_*1*_, ..., *y*_*n*_, is proportional to the likelihood, and the probability density of the prior distribution of *α*, *β *and *σ*^2^. The MCMC method is employed using the WinBUGS software to obtain the required summaries of the posterior distributions of *α*, *β *and *σ*^2^. For given Y_0_, the posterior distribution of

(8) $$\log_{10}(X_{0})=\frac{Y_{0}-\alpha }{\beta },$$

can be easily used to obtain summary statistics, such as mean, median and 95% BCI, for the unknown DNA concentration log_10 _(X_0_).

### Developing a Master Calibration Curve from Multiple qPCR Experiments

Calibration curves from several independent runs are pooled together to obtain a master calibration curve. A hierarchical Bayesian model is used to allow for run to run variability in estimating a master calibration curve. As several calibration curves are produced in this study, the slope and intercept parameters of the calibration curves are allowed to vary from run to run in developing a master calibration curve. Equation (4) is modified to allow for run to run variability in the intercept and slope parameters. The general form of the regression model is given by:

(9) $$\begin{array}{l} {\text{Y}}_{\text{ijk}}\sim \text{N}(\mu_{\text{ij}},\sigma_{\text{i}}^{2}),\\ \mu_{\text{ij}}=\alpha_{\text{i}}+\beta_{\text{i}}*\log_{10}(X_{\text{ij}}),\\ \alpha_{\text{i}}\sim \text{N}(\bar{\alpha },\sigma_{\text{a}}^{2}),\\ \begin{array}{cccc} \beta_{\text{i}}\sim \text{N}(\bar{\beta },\sigma_{\text{b}}^{2}), & \text{k}=1,2,..{\text{n}}_{\text{ij}}, & \text{i}=1,2...\text{n}; & \text{j}=1,2,..\text{m}; \end{array} \end{array}$$

where, Y_ijk _is the kth Ct measurement of jth copy number and ith run, X_ij _is the jth copy number for ith run, *α*_i _and *β*_i _are regression coefficients for ith run, $\sigma_{\text{i}}^{2}$ is the random error variance of the ith calibration curve, $\bar{\alpha }$ and $\bar{\beta }$ are the overall regression coefficients, combining information from all runs. The following diffused prior distributions are used to estimate the model parameters:

$$\begin{array}{l} \bar{\alpha },\bar{\beta }\sim \text{N}(0,10^{4})\\ \begin{array}{cc} \sigma_{\text{a}}^{2},\sigma_{\text{b}}^{2},\sigma_{\text{i}}^{2}\sim \text{Inv}\text{. Gamma}(.001,.001) & \text{i}=1,2...\text{n}. \end{array} \end{array}$$

We also used the prior distribution recommended by DuMouchel for *σ*_a _and *σ*_b_, which is based on the harmonic mean of the estimated variances of the intercepts and slopes of individual calibration curves [23]. DuMouchel priors for *σ*_a _and *σ*_b _are given by:

(10) $$\begin{array}{c} \sigma_{\text{a}}\sim \frac{U(1-U)}{\sqrt{(\sum_{1}^{n}1/\mathrm{var}({\hat{\alpha }}_{i}))/n}}\\ \sigma_{\text{b}}\sim \frac{U(1-U)}{\sqrt{(\sum_{1}^{n}1/\mathrm{var}({\hat{\beta }}_{i}))/n}} \end{array}$$

where, U stands for the standard Uniform distribution U(0,1) and var(${\hat{\alpha }}_{i}$) and var(${\hat{\beta }}_{i}$) are respectively the estimated variances of the least squares estimates of *α*_i _and *β*_i_. The results obtained using the DuMouchel and Gamma priors for *σ*_a _and *σ*_b _are very similar. A MCMC simulation method was used to estimate the model parameters via WinBUGS software. Convergence diagnostics of Markov Chain draws from the posterior distributions of the parameters were checked using trace plots, auto-correlation plots, and Gelman and Rubin diagnostics [24,25], and found to be satisfactory (data not shown).

**Figure 1:**
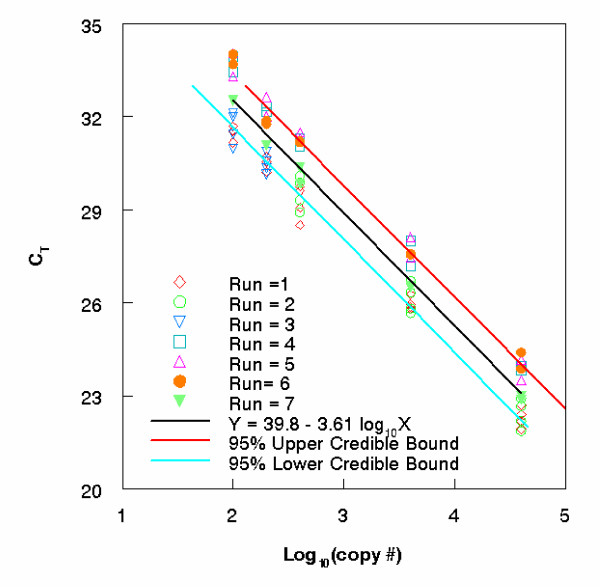
**Developing a master calibration curve**. Seven independent calibration data sets from Entero2 (genomic type) are used to obtain a single master calibration curve and the corresponding 95% BCI.

**Figure 2:**
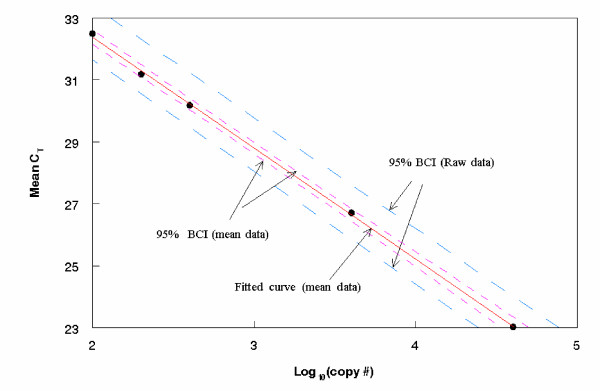
**Comparing 95% Bayesian credible intervals**. The mean data (plotted) from seven independent runs for Entero2 assay (genomic type) is used to generate the fitted curve and the 95% BCI. The corresponding 95% BCI for the row data is also included for comparison purposes.

For given Y_0_, by requesting the posterior distribution of

(11) $$\log_{10}({\text{X}}_{0})=\frac{{\text{Y}}_{0}-\bar{\alpha }}{\bar{\beta }}$$

from the WinBUGS program, one can easily obtain summary statistics, such as mean, median and 95% credible interval for the mean of log_10 _(X_0_). Replacing $\bar{\alpha }$ by *α*_i _and $\bar{\beta }$ by *β*_i _in equation (10), we get the posterior distribution for the ith run (see Additional file 1). The estimated mean copy number corresponding to different C_T _measurements are plotted in Figure 1 for Entero2 genomic type (seven independent runs). Notice that the 95% upper and lower credible bounds and the fitted curve are for the copy number (in log base 10) in Figure 1. For comparison purposes, the averaged concentration data is used to obtain a fitted master curve and 95% BCI, for mean DNA concentration, and these are given in Figure 2 along with the corresponding 95% BCI using the raw data. It is better to use the raw data (than the averaged data) as it allows accounting for the within and between run variations in constructing credible interval for DNA concentration. Allowing for these (additional) variations would lead to more realistic and wider confidence intervals. Consequently, the 95% BCI is wider for the raw data than for the averaged data.

### Fitting a Genomic DNA Calibration Curve using Three Independent qPCR Assays

In real time qPCR studies using absolute standards, usually a calibration curve is developed to estimate an unknown DNA concentration. Typically, either plasmid or genome type calibration curves can be developed for a given assay. But, there are instances where PCR assays designed to target genomic DNA sequences must rely on plasmid derived absolute DNA standards to generate calibration curves such as PCR assays targeting genes from uncultivated microorganisms. qPCR assays that rely on plasmid absolute DNA standards to estimate genomic DNA concentrations from unknown samples must either assume that there is no difference in the amplification efficiencies between these two DNA types or estimate differences and account for this uncertainty in respective calibration curve statistics. A simulation method to estimate the genomic DNA type calibration curve for the assay HF183 using both plasmid and genomic DNA type curves of Btheta, Entero1 and Entero2 assays is discussed in this section.

The model described in equation (9) was applied to all four assays with an additional suffix. In the following model, this suffix is set to 1 (for Btheta, plasmid type), 2 (for Btheta, genome type), 3 (for Entero1, plasmid type), 4 (for Entero1, genome type), 5 (for Entero2, plasmid type), 6 (for Entero2, genome type) and 7 (HF183, plasmid type).

(12) $$\begin{array}{l} {\text{Y}}_{\text{ijkl}}\sim \text{N}(\mu_{\text{ijl}},\sigma_{\text{il}}^{2}),\\ \mu_{\text{ijl}}=\alpha_{\text{il}}+\beta_{\text{il}}*\log_{10}(X_{\text{ijl}}),\\ \alpha_{\text{il}}\sim \text{N}({\bar{\alpha }}_{l},\sigma_{\text{al}}^{2}),\\ \begin{array}{cccc} \beta_{\text{il}}\sim \text{N}({\bar{\beta }}_{l},\sigma_{\text{bl}}^{2}), & \text{k}=1,2,..{\text{n}}_{\text{ijl}}, & \text{i}=1,2...\text{n}; & \begin{array}{cc} \text{j}=1,2,..\text{m}; & \text{l}=1,2,..7. \end{array} \end{array} \end{array}$$

The following priors are used to estimate the model parameters:

$$\begin{array}{l} {\bar{\alpha }}_{l},{\bar{\beta }}_{l}\sim \text{N}(0,10^{4})\\ \begin{array}{cc} \sigma_{\text{al}},\sigma_{\text{bl}}\sim \text{DuMouchel} & \text{l}=1,2...7 \end{array}\\ \begin{array}{ccc} \sigma_{\text{il}}^{2}\sim \text{Inv}\text{. Gamma}(.001,.001), & \text{i}=1,2...\text{n}; & \text{l}=1,2..7. \end{array} \end{array}$$

where the DuMouchel priors for *σ*_a1 _and *σ*_b1 _are based on the least square estimates of *α*_il _and *β*_il_, respectively (see equation (10)).

**Figure 3:**
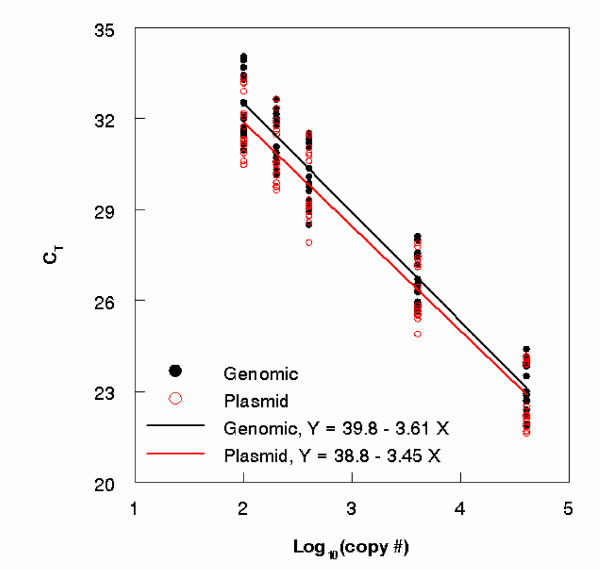
**Genomic versus plasmid DNA standard curves**. Fitted curves derived from seven independent runs for both genomic and IAC plasmid DNA standards for the Entero2 qPCR assay. ANCOVA indicated a significant difference in slope (p < .05).

To test for potential differences between genomic and plasmid DNA standard curves, overall fitted curves representing seven to eight independent runs for genomic DNA standards with a 6FAM labeled probe and plasmid DNA standards with a TET labeled probe for three FIB assays (Btheta, Entero1 and Entero2) were compared using analysis of covariance (ANCOVA) test. A significant difference between genomic and plasmid DNA type approaches was observed in slopes for Btheta (p = .0088) and Entero2 (p = .0393, see Figure 3) assays. Thus the assumption that there are no differences between respective genomic and plasmid DNA types held for only one of the three assays. For Btheta, Entero1, and Entero2, the difference between the genomic DNA type calibration curve intercept and the plasmid DNA type calibration curve intercept are respectively ${\bar{\alpha }}_{2}-{\bar{\alpha }}_{1},{\bar{\alpha }}_{4}-{\bar{\alpha }}_{3}$ and ${\bar{\alpha }}_{6}-{\bar{\alpha }}_{5}$. The respective differences between the slopes are ${\bar{\beta }}_{2}-{\bar{\beta }}_{1},{\bar{\beta }}_{4}-{\bar{\beta }}_{3}$ and ${\bar{\beta }}_{6}-{\bar{\beta }}_{5}$. The fitted genomic and plasmid DNA calibration curves indicated the least variability in posterior mean slope and intercept differences for Entero1 and the most for Entero2 (see Additional file 2, output) suggesting that differences between plasmid and genomic DNA curves can vary from one PCR assay to another. As the genomic DNA calibration curve is not available for HF183, we used all three FIB assays to modify the plasmid DNA curve of HF183 to estimate variation between the known plasmid DNA curve and the uncharacterized genomic DNA curve. The intercept and slope of HF183 genome type calibration curve was estimated by adding the corresponding mean differences from the plasmid and genome type calibration curves of Btheta, Entero1, and Entero2 to the plasmid type curve of HF183. Thus, the intercept ${\bar{\alpha }}_{8}$ and slope β¯8 of HF183 genome type calibration curve are given by:

(13) $$\begin{array}{l} {\bar{\alpha }}_{8}={\bar{\alpha }}_{7}+[({\bar{\alpha }}_{2}-{\bar{\alpha }}_{1})+({\bar{\alpha }}_{4}-{\bar{\alpha }}_{3})+({\bar{\alpha }}_{6}-{\bar{\alpha }}_{5})]/3\\ {\bar{\beta }}_{8}={\bar{\beta }}_{7}+[({\bar{\beta }}_{2}-{\bar{\beta }}_{1})+({\bar{\beta }}_{4}-{\bar{\beta }}_{3})+({\bar{\beta }}_{6}-{\bar{\beta }}_{5})]/3 \end{array}$$

**Figure 4:**
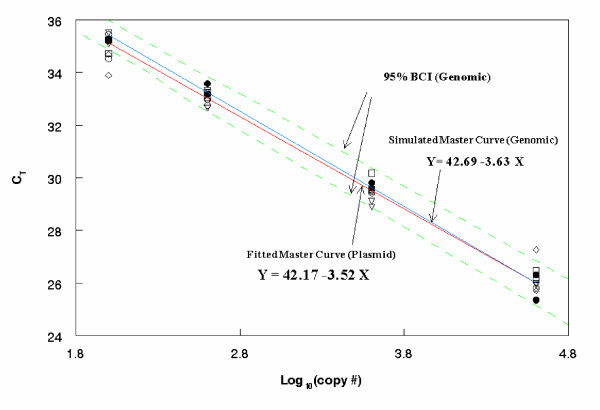
**Estimating a genomic master calibration curve**. Mean differences of intercepts and slopes between genome type and plasmid type master calibration curves of three assays(Btheta, Entero1& Entero2) are added to the plasmid master calibration curve (five runs) to generate a simulated genomic master calibration curve for HF183.

By utilizing the posterior distributions of ${\bar{\alpha }}_{8}$ and β¯8 from the WinBUGS program, one can estimate the slope and intercept parameters of the genomic type calibration curve for Entero2 (See Additional file 2). Figure 4 gives the fitted plasmid and simulated genome master calibration curves for HF183 with a 95% BCI.

### Estimating DNA Concentration from a Modified Master Calibration Curve

The modified master calibration curve for HF183 with intercept and slope parameters ${\bar{\alpha }}_{8}$ and β¯8 was used to obtain estimate DNA concentrations from recreational water samples (see Additional file 2). For given Y, the posterior distribution of log_10_(X_0_), where

(14) $$\begin{array}{c} \log_{10}({\text{X}}_{0})=(\text{Y}-{\bar{\alpha }}_{8})/{\bar{\beta }}_{8}\\ =\frac{Y-\{{\bar{\alpha }}_{7}+[({\bar{\alpha }}_{2}-{\bar{\alpha }}_{1})+({\bar{\alpha }}_{4}-{\bar{\alpha }}_{3})+({\bar{\alpha }}_{6}-{\bar{\alpha }}_{5})]/3\}}{{\bar{\beta }}_{7}+[({\bar{\beta }}_{2}-{\bar{\beta }}_{1})+({\bar{\beta }}_{4}-{\bar{\beta }}_{3})+({\bar{\beta }}_{6}-{\bar{\beta }}_{5})]/3} \end{array}$$

was used to estimate the mean, standard deviation and 95% credible intervals for unknown DNA concentration. Estimates for four unknown samples are given in the output section of Appendix B (see Additional file 2). Even though log_10_(X_0_) is a non-linear function of the parameters ${\bar{\alpha }}_{1},...{\bar{\alpha }}_{7};{\bar{\beta }}_{1},...{\bar{\beta }}_{7}$, the Bayesian MCMC simulation method can be easily applied to estimate X_0_. To evaluate the impact of prior distributions, Uniform prior was used for each of *σ*_a1_and *σ*_b1 _(l = 1...7). No apparent difference was seen in the resulting mean, median or 95% BCI of the two posterior distributions of any of the model parameters (data not shown).

## Conclusion

We employed a Bayesian approach for the estimation of DNA concentrations from environmental samples using absolute standard curves generated by real-time qPCR. Our approach allowed us to account for uncertainty from multiple sources such as experiment-to-experiment variation, variability between replicate measurements, as well as uncertainty introduced when employing calibration curves generated from absolute plasmid DNA standards. The Bayesian approach also allowed for the estimation of model parameters from multiple models simultaneously unlike stepwise progression of estimates typically used in real-time PCR calibration calculations. The flexible modeling capability of the Bayesian approach was ideal for real-time qPCR assays that rely on absolute plasmid DNA standards for quantification and this method should be applicable over a wide range of study designs.

## Methods

### Sample collection and DNA extraction

Select individual fecal and recreational water samples were collected as previously described [26]. All DNA extractions were performed with the FastDNA Kit for Soils (Q-Biogene; Carlsbad, CA) [26].

### Genomic DNA standard preparations from pure bacterial cultures

American Type Culture Collection (ATCC) bacterial strains were used to prepare genomic DNA calibration standards. *E. faecalis *(ATCC #29212) was cultured as previously described [27]. *B. thetaiotaomicron *(ATCC # 29741) cells were grown in chopped meat carbohydrate broth (Remel, Lenexa, KS) according to manufacturer's instructions. Both cultures were harvested by centrifugation at 8,000 × *g *for 5 min, washed twice using sterile phosphate buffered saline (Sigma, St. Louis, MO) and stored in aliquots at -40°C. Cell concentrations of each organism in the final washed suspensions were determined by bright field microscopy at 40× magnification in disposable hemocytometer chambers (Nexcelom Bioscience, #CP2-002, Lawrence, MA). DNA was isolated from the cell suspensions using a bead beating extraction approach [27] and incubated for one hour at 37°C with 0.017 *μ*g/*μ*l RNase A (Gentra Systems, USA). DNA purification was performed using a silica column adsorption kit (DNA-EZ, GeneRite, Kendall Park, NJ.). DNA concentrations of cell extracts were determined by spectrophotometric absorbance readings at 260 nm (A_260_) and purity of the DNA preparations was determined by A_260_/A_280 _ratios.

### Plasmid DNA standard preparation

**Figure 5:**
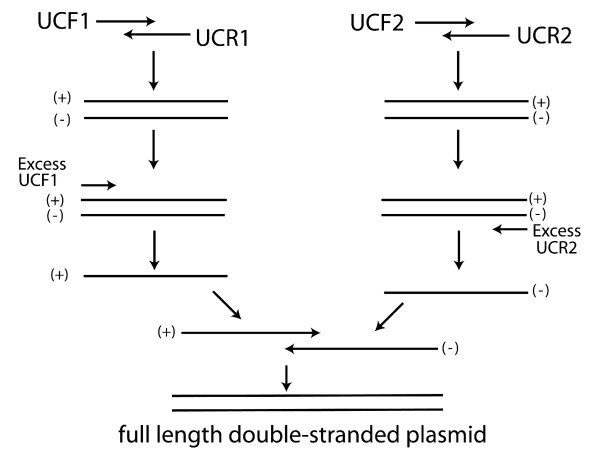
To build the plasmid absolute standard construct, long oligonucleotides (~80 bp, Table 1) containing multiple primer binding sequences were designed such that their 3' ends overlapped. The two overlapping fragments were then combined into a single DNA molecule using overlap extension PCR [29]. Each partially overlapping fragment generated from the initial overlap extension was combined by a second overlap extension into a single full-length DNA construct.

A single plasmid containing a single site for hybridization of a unique TaqMan^® ^TET labeled probe sequence flanked by PCR primer binding sites for all four qPCR assays was developed using overlap extension PCR [Figure 5, [28]]. To build the plasmid construct, long oligonucleotides (> 100 bp, Table 1) containing multiple primer sequences [29] were designed such that their 3' ends overlapped. Overlapping fragments were then combined into a single DNA molecule using a two step overlap extension PCR, i.e. the partially overlapping products of two initial overlap extension PCR experiments were combined by a second overlap extension PCR. The plasmid construct was then inserted into a pCR4^® ^TOPO plasmid vector (Invitrogen) and the resulting recombinant plasmid was purified from transformed *E. coli *cell cultures using a Qiagen Plasmid Purification Kit (Qiagen, Valencia, CA). Plasmid DNA was linearized by a Not1 restriction digestion (New England BioLabs, Beverly, MA), quantified with a NanoDrop ND-1000 UV spectrophotometer (NanoDrop Technologies), and diluted in 10 mM Tris, 0.1 mM EDTA, pH 8.0 to generate samples ranging from approximately 100 to 4 × 10^4 ^molecules. Dilutions were stored in TE buffer (10 mM Tris, 0.1 mM EDTA, pH 8.0) in single use aliquots.

**Table 1:** Oligonucleotides and probe used in study.

	**Sequence 5' → 3'**	**Reference**
Btheta	*F*-CGTTCCATTAGGCAGTTGGT	[30]
	*R*-ACACGGTCCAAACTCCTACG	
Entero1	*F*-AGAAATTCCAAACGAACTTG	[31]
	*R*-AATGATGGAGGTAGAGCACTGA	
Entero2	*F*-GAGGACCGAACCCACGTA	[32]
	*R*-CAGTGCTCTACCTCCATCATT	
HF183	*F*-ATCATGAGTTCACATGTCCG	[32,33]
	*R*-CCGTCATCCTTCACGCTACT	
UC1F1	CCGTCATCCTTCACGCTACTGAGGACCGAACCCACGTACCCTTC	This Study
	AGTGCCGCAGTCGTTCCATTAGGCAGTTGGTGAGAAA	
UC1R1	CCTGCCGTCTCGTGCTCCTCAAACGCTTCTTAGTCAGGTACCGT	
	CAAGTTCGTTTGGAATTTCTCACCAACTGCCTAATG	
UC1F2	TGAGGAGCACGAGACGGCAGGAACCTTCCTCTCAGAACCCCAATG	
	ATGGAGGTAGAGCACTGACACGGTCCAAACTCCTA	
UC1R2	GATCATGAGTTCACATGTCCGCGTCGCAGGATGTCAAGACAGTA	
	AATCCGGATAACGCTCGTAGGAGTTTGGACCGTGTCA	
UC1	[TET] CCTGCCGTCTCGTGCTCCTCA [TAMRA]*	[35]

An * indicates that the TaqMan^® ^probe was modified from the previously reported UT Probe [35].

### Quantitative real-time PCR

Four qPCR assays were used in this study including HF183, Btheta, Entero1, and Entero2 (Table 1) [30-33]. Amplification was performed in a 7900 HT Fast Real Time Sequence Detector (Applied Biosystems) with default thermal cycle conditions. Reaction mixtures (25 *μ*l) contained 1X TaqMan^® ^Universal PCR Master Mix with AmpErase^® ^uracil-N-glycosylase (UNG, Applied Biosystems), 0.2 mg/ml bovine serum albumin (Sigma), 1 *μ*M of each primer, 80 nM FAM™ or TET™ labeled TaqMan^® ^probe (Applied Biosystems), and either 2 ng genomic DNA (unknown samples) or 100 to 4 × 10^4 ^target gene copies (plasmid or purified genomic DNA). All reactions were performed in triplicate. Data was initially analyzed with Sequence Detector Software (Version 2.2.2) at a threshold determination of 0.03. Threshold cycle (C_T_) values were exported to Microsoft Excel for further statistical analysis.

### Data analysis

An analysis of Covariance (ANCOVA) model was used to compare the overall mean intercept and slope of genome standard curves with the corresponding overall mean intercept and slope of the corresponding plasmid standard curves. ANCOVA tests were performed using SAS programs (Cary, North Carolina) with the following procedure "PROC MIXED" [34]. Markov Chain Monte Carlo (MCMC) simulation method was used to obtain single, master, and modified calibration curves. Summaries of the posterior distribution such as the mean and the percentiles were used as point and interval estimates of unknown parameters of interest. The software WinBUGS versions 1.4.1 [22] was used to perform all simulations.

## Authors' contributions

MS, OCS, and RAH contributed to development of the methodology. MV and SS performed all real-time qPCR experiments.

## Supplementary Material

Additional File 1 Appendix A. This file contains the BUGS code to generate a master calibration curve. Click here for file

Additional File 2 Appendix B. This file contains the BUGS code to generate a calibration curve for HF183 genome type. The output section provides the summary statistics for the model parameters. Click here for file
