# Supplementary material for: A Bayesian method for calculating real-time quantitative PCR calibration curves using absolute plasmid DNA standards
Source: BMC Bioinformatics. 2008 Feb 25;9:120. doi: 10.1186/1471-2105-9-120 (PMC2292693; doi:10.1186/1471-2105-9-120)
Supplement: Additional File 1 — Appendix A. This file contains the BUGS code to generate a master calibration curve. [file 1471-2105-9-120-S1.pdf]

Appendix A

```
# generating a master calibration curve
model
    {
        for( i in 1 : n ) {
            Y[i] ~ dnorm(mu[i],tau[run[i]])
            mu[i] <- alpha[run[i]]  + beta[run[i]] * (log(X[i]) )/log(10)  }
        for (i in 1 : m) {
            beta[i]  ~ dnorm(beta_bar,taub)
           alpha[i] ~ dnorm(alpha_bar, taua)   }
# non_informative priors
# σ2 [i] =1/tau[i],  σ2a =1/taua , σ2b =1/taub
        for ( i in 1:m)  {
          tau[i] ~ dgamma(.001,.001)  }
         taua ~ dgamma(0.001,0.001)
         taub ~ dgamma(0.001,0.001)
        alpha_bar ~ dnorm(0,1.0E-4)
        beta_bar ~ dnorm(0,1.E-4)
#predict copy no. (= log10(X0)) for given ct
        for (i in 1:6)  {
            copy[i]  <- (ct[i] -alpha_bar)/beta_bar    }
    }
Data
list( n=50, m=5)
run[]   X[]      Y[]

1       40000    22.30
1       40000    22.16
.         .        .
.         .        .

1       400      29.04
.         .        .
.         .        .

5       100      31.84
END;
ct[]
22
24
26
28
30
32
END;
```
