# Supplementary material for: A Bayesian method for calculating real-time quantitative PCR calibration curves using absolute plasmid DNA standards
Source: BMC Bioinformatics. 2008 Feb 25;9:120. doi: 10.1186/1471-2105-9-120 (PMC2292693; doi:10.1186/1471-2105-9-120)
Supplement: Additional File 2 — Appendix B. This file contains the BUGS code to generate a calibration curve for HF183 genome type. The output section provides the summary statistics for the model parameters. [file 1471-2105-9-120-S2.pdf]

Appendix B

```
# generating a calibration curve for HF183 genome type
model
        {
 #  genome and plasmid calibration curves for Assays Btheta, Entero1 and Entero2
           for( i in 1 : n ) {
                CT_genome[i] ~ dnorm(mu1[i],tau_g[set[i]])
                mu1[i] <- alpha_g[set[i]]  + beta_g[set[i]] * (log(Copy[i]) )/log(10)
                CT_plasmid[i] ~ dnorm(mu2[i],tau_p[set[i]])
                mu2[i] <- alpha_p[set[i]]  + beta_p[set[i]] * (log(Copy[i]) )/log(10)   }
#  plasmid calibration curve for  assay HF183
          for (i in 1:n4) {
               CT_plasmid4[i] ~ dnorm(mu4[i],tau_p4[run4[i]])
               mu4[i] <- alpha_p4[run4[i]]  + beta_p4[run4[i]] * (log(Copy4[i]) )/log(10)
#  8 independent slope and intercept  parameters  for Btheta (plasmid and genome)
        for (i in 1 : 5) {
             beta_g[i]  ~ dnorm(bg[1],taubg[1])
             alpha_g[i] ~ dnorm(ag[1], tauag[1])
             beta_p[i]  ~ dnorm(bp[1],taubp[1])
             alpha_p[i] ~ dnorm(ap[1], tauap[1])   }
 #  8 independent slope and intercept parameters for Entero1 (plasmid and genome)
           for (i in 6 : 10) {
                beta_g[i]  ~ dnorm(bg[2],taubg[2])
               alpha_g[i] ~ dnorm(ag[2], tauag[2])
               beta_p[i]  ~ dnorm(bp[2],taubp[2])
               alpha_p[i] ~ dnorm(ap[2], tauap[2])   }
 #  7 independent slope and intercept parameters for Entero2 (plasmid and genome)
         for (i in 11 : 15) {
               beta_g[i]  ~ dnorm(bg[3],taubg[3])
              alpha_g[i] ~ dnorm(ag[3], tauag[3])
              beta_p[i]  ~ dnorm(bp[3],taubp[3])
              alpha_p[i] ~ dnorm(ap[3], tauap[3])   }
#  5 independent slope and intercept parameters  for HF183 (only plasmid)
         for (i in 1 : 7) {
              beta_p4[i]  ~ dnorm(bp4,taubp4)
              alpha_p4[i] ~ dnorm(ap4, tauap4) }
#  non-informative priors
         for (i in 1:m)  {
             tau_g[i] ~ dgamma(.001,.001)
             tau_p[i] ~ dgamma(.001,.001) }
        for (i in 1 : 3) {
             ap[i] ~ dnorm(0,1.0E-4)
             bp[i] ~ dnorm(0,1.E-4)
             ag[i] ~ dnorm(0,1.E-4)
             bg[i] ~ dnorm(0,1.0E-4)
```

```
                 tau_p4[i] ~ dgamma(.001,.001)  }
                  ap4 ~ dnorm(0,1.0E-4)
                  bp4 ~ dnorm(0,1.E-4)
#   Dumouchal priors
              for (i in 1 : 3) {
                  U_1[i] ~ dunif(0, 1)
                  sqrvarbp[i] <- s_slope_p[i] * (1 - U_1[i]) / U_1[i]
                  varbp[i] <- sqrvarbp[i] * sqrvarbp[i]
                  taubp[i] <- 1/varbp[i]
                  U_2[i] ~ dunif(0, 1)
                  sqrvarbg[i] <- s_slope_g[i] * (1 - U_2[i]) / U_2[i]
                  varbg[i] <- sqrvarbg[i] * sqrvarbg[i]
                  taubg[i] <- 1/varbg[i]
                 U_3[i] ~ dunif(0, 1)
                 sqrvarap[i] <- s_int_p[i] * (1 - U_3[i]) / U_3[i]
                 varap[i] <- sqrvarap[i] * sqrvarap[i]
                 tauap[i] <- 1/varap[i]
                 U_4[i] ~ dunif(0, 1)
                 sqrvarag[i] <- s_int_g[i] * (1 - U_4[i]) / U_4[i]
                varag[i] <- sqrvarag[i] * sqrvarag[i]
                 tauag[i] <- 1/varag[i]         }
                 U1 ~ dunif(0, 1)
                sqrvarbp4  <-  s_slope_p4 * (1 - U1) / U1
                varbp4  <-  sqrvarbp4 * sqrvarbp4
                 taubp4 <-  1/varbp4
                 U2 ~ dunif(0, 1)
                 sqrvarap4 <- s_int_p4 * (1 – U2) / U2
                 varap4 <- sqrvarap4 * sqrvarap4
                 tauap4 <- 1/varap4
#   estimating the difference in slope and intercept for Btheta, Entero1 and Entero2
            for (i in 1 : 3) {
                diff_slope[i] <-  bg[i] - bp[i]
                diff_int[i] <- ag[i] -  ap[i]  }
#   estimating the mean difference in slope and intercept (to be used in HF183)
            d_slope <- mean(diff_slope[])
            d_int <- mean(diff_int[])
#   estimating the genome intercept and slope for HF183
            genome4_int <-  ap4 + d_int
            genome4_slope <-  bp4 + d_slope
#   estimatinf the log10 copy number for HF183
            for (i in 1:np4)  {
                lcopy4[i]  <-  (ct4[i] - genome4_int)/genome4_slope  }
             }
```

Data

list(n=239, m=23, n4=40,np4=4, s_slope_p=c(0.075104,0.095792,0.107489),
s_slope_g=c(0.085519 , 0.066039 , 0.098919),

```
s_int_p=c(0.238018,0.303586,0.346371),   s_int_g=c( 0.271027,0.210087, 0.315319),
s_slope_p4=0.123962, s_int_p4=0.415432))
run[]    Copy[]   CT_genome[]    CT_plasmid[]
1         100       32.10291          31.25919
1         100       32.221012         31.16806
1          200      30.735775         30.46597
1          200      31.085876         30.20037
.
.
23        100       32.513058         31.132364
23        100       32.539833         31.328972
END;
run4[]   Copy4[]      CT_plasmid4[]
1         40000         27.261871
1         40000         25.704222.
.
.
5         100            35.2057
5         100           35.28721
END;
# Unknown samples
ct4[]
33.49
32.11
32.05
39.46
END;
```

Output

Assay	Type	Parameter	node	Mean	St. dev	95% LCB	95% UCB
**Btheta**	Plasmid	intercept	alpha_p[1]	38.83	0.36	38.12	39.55
		slope	beta_p[1]	-3.45	0.03	-3.51	-3.38
	Genome	intercept	alpha_g[1]	39.37	0.27	38.84	39.90
		slope	beta_g[1]	-3.60	0.04	-3.67	-3.53
		intercept diff.	diff_int[1]	0.54	0.45	-0.36	1.44
		slope diff.	diff_slope[1]	-0.15	0.05	-0.26	-0.05
**Entero1**	Plasmid	intercept	alpha_p[2]	38.36	0.14	38.08	38.64
		slope	beta_p[2]	-3.45	0.04	-3.53	-3.36
	Genome	intercept	alpha_g[2]	38.42	0.08	38.26	38.58
		slope	beta_g[2]	-3.47	0.03	-3.52	-3.42
		intercept diff.	diff_int[2]	0.06	0.17	-0.27	0.39
		slope diff.	diff_slope[2]	-0.03	0.05	-0.13	0.07
**Entero2**	Plasmid	intercept	alpha_p[3]	38.81	0.46	37.90	39.71
		slope	beta_p[3]	-3.46	0.05	-3.56	-3.36
	Genome	intercept	alpha_g[3]	39.75	0.41	38.93	40.58
		slope	beta_g[3]	-3.61	0.05	-3.71	-3.53
		intercept diff.	diff_int[3]	0.94	0.61	-0.28	2.15
		slope diff.	diff_slope[3]	-0.16	0.07	-0.29	-0.01
**All 3 assays**		mean int. diff.	d_int	0.52	0.26	-0.09	1.03
		mean slope diff.	d_slope	-0.11	0.03	-0.18	-0.04
**HF183**	Plasmid	intercept	alpha_p4	42.17	0.23	41.70	42.62
		slope	beta_p4	-3.52	0.07	-3.66	-3.37
	**Genome (simulated)**	intercept	genome4_int	42.69	0.35	41.99	43.37
		slope	genome4_slope	-3.63	0.08	-3.79	-3.46
	River **(unknown)**	Log_{10} (copy #)	lcopy4[1]	2.54	0.08	2.38	2.69
			lcopy4[2]	2.92	0.08	2.76	3.08
			lcopy4[3]	2.93	0.08	2.78	3.09
	Marine**(unknown)**		lcopy4[4]	0.89	0.09	0.72	1.06
